# Prognostic determinants in surgical critial patients undergoing emergency surgery for Stage III or higher colorectal cancer

**DOI:** 10.1007/s00423-025-03653-4

**Published:** 2025-02-22

**Authors:** Hyun Ho Kim, Sanguk Hwang, Jinbeom Cho

**Affiliations:** 1 Department of Surgery, Bucheon St. Mary’s Hospital, College of Medicine, The Catholic University of Korea, Seoul, Republic of Korea; 2 Department of Artificial Intelligence, The Catholic University of Korea, Bucheon, Republic of Korea

**Keywords:** Colorectal cancer, Emergency, Surgery, Prognosis

## Abstract

**Purpose:**

Emergency surgery in patients with colorectal cancer (CRC) is associated with elevated mortality and morbidity compared to elective operations. This study was conducted to identify the factors influencing both short and long term outcomes in emergent CRC operations, particularly in critically ill patients.

**Method:**

This single center retrospective analysis focuses on patients with stage III or higher CRC who underwent emergency surgery and were admitted to the intensive care unit postoperatively.

**Results:**

Among 64 patients, 46 presented with generalized peritonitis due to free perforation. Non-survivors at 90 days had a higher incidence of preoperative shock (53.3% vs. 4.1%, *P =* 0.000), elevated perioperative Sequential Organ Failure Assessment scores (*P =* 0.000; *P =* 0.013), and fewer retrieved lymph nodes (LN) (*P =* 0.010). Multivariate analysis identified LNs retrieval as a significant predictor of 90-day mortality (AUC = 0.727). For overall survival, younger age, lower American Society of Anesthesiologists (ASA) physical status, absence of metastasis, adjuvant chemotherapy (CTx), and lower LN ratio (LNR) were associated with improved outcomes. Multivariate analysis showed ASA physical status and adjuvant CTx as significant predictors. In predicting 3-year recurrence (51% of patients), the Random Forest model achieved 65% accuracy. Age and LNR were major predictors, with 0.01 unit increase in LNR raising recurrence risk by 1.025-fold and each additional year of age by 1.035-fold.

**Conclusion:**

The number of retrieved LNs was identified as a predictor of 90 day survival, ASA physical status and adjuvant CTx were identified as prognostic factors for overall survival, and age and the LNR were found to be predictors of disease recurrence within three years.

## Introduction

Colorectal cancer (CRC) is a major global health concern, accounting for over 1.9 million new cases and approximately 935,000 deaths worldwide in 2020 [1]. Despite advancements in screening and early detection, up to 30% of CRC patients still present with advanced disease at diagnosis, often accompanied by complications such as bowel obstruction (15–29%) and perforation (3–8%) [2, 3]. These emergency presentations necessitate urgent surgical interventions, which are associated with worse clinical outcomes, including higher perioperative morbidity, mortality, and increased recurrence rates compared to elective surgeries [3, 4]. In general, meticulous preoperative work-up and staging are vital for CRC surgery, and if definitive surgery is deemed unfeasible, options such as self-expandable metal stents or a proximal decompressing colostomy may be considered [5, 6]. However, in certain scenarios, emergent operation is mandatory, such as when free colorectal perforation leads to generalized peritonitis and a heightened risk of septic shock, when cancer obstruction is accompanied by localized perforation, or when stenting or decompressing stoma cannot be employed to manage complete obstruction. Under these circumstances, surgeons often face a dilemma between performing only the minimal procedure to stabilize a critically ill patient and performing a more comprehensive oncologic resection. To address these challenges, this study aims to identify the factors influencing both surgery-related short-term outcomes and oncologic long-term outcomes in emergency CRC operation, thereby offering perspectives on appropriate surgical approaches and postoperative management.

## Methods

This single center retrospective study was approved by the Institutional Review Board of our institution (HC24RISI0082). We included patients treated at single center between January 2010 and December 2021 who met all of the following inclusion criteria: (1) patients who underwent emergency surgery after an established diagnosis of CRC, or in whom CRC was detected during an emergency operation performed for a different indication.; (2) patients confirmed to have stage III or higher CRC based on pathological examination; (3) patients who required definitive surgical resection of the lesion, as stenting or a decompressing colostomy alone was insufficient to resolve their underlying clinical issues.; and (4) patients who required level II or higher critical care in the intensive care unit postoperatively. Patients who underwent emergent surgery for a newly arising CRC after having previously completed CRC treatment, and those in whom the main tumor was deemed unresectable and therefore received only bypass or diversion for palliation, were excluded from the study.

Throughout the study period, a consistent surgical team oversaw decision-making, surgical interventions, and patient management in accordance with a standardized protocol. The patients’ clinical and laboratory data were analyzed, with operative outcomes defined by 90-day survival. Based on a review of the literature [7, 8], the number of retrieved lymph nodes (LN) was categorized into two groups: 12 or more, and fewer than 12. LN ratio (LNR) was determined as the proportion of positive LNs relative to the total number of LNs retrieved. Clavien-Dindo (CD) grades IIIa, IIIb, IVa, and IVb were included as postoperative complications [9]. Although CD grades I and II are typically regarded as minor complications, due to the retrospective nature of this study, which relied on medical record reviews, it was not possible to distinguish between these two categories in detail, leading to their inclusion in the non-complicated group. All included patients presented with stage III or higher disease. Consequently, adjuvant CTx was administered whenever feasible based on the patient’s postoperative performance status. However, for those with compromised performance, CTx could not be administered. Adjuvant CTx was defined as being administered in cases where the patient either completed the cycle post-surgery or was confirmed to have recurrence during the first-line CTx, prompting the initiation of second-line CTx. If the patient did not complete the first-line CTx cycle, they were considered as not having received adjuvant CTx. The oncologic outcomes were categorized into overall survival (OS) and disease-free survival (DFS). Given that the follow-up periods varied among patients, OS was analyzed based on the survival days for each patient, without setting a specific end-point, and DFS was included as a categorical variable, defined by whether recurrence occurred within the 3-year period, rather than as a continuous variable. During the postoperative follow-up period, regular imaging studies were conducted. Recurrence was defined as either the detection of new lesions on these imaging studies or disease progression.

### Statistical analysis

Summary statistics are presented as frequencies and percentages for categorical variables, and as means ± standard deviations for continuous variables. Normality was evaluated using the Shapiro-Wilk test. Categorical variables were analyzed using either the chi-square test or Fisher’s exact test, while continuous variables were assessed using the t-test or the Wilcoxon test to compare observed differences. Statistically significant variables identified through multivariate logistic regression were further examined by calculating the area under the receiver operating characteristic (ROC) curve (AUC) to assess their predictive value. For the analysis of OS, ANOVA was applied to categorical variables, while linear regression was used for continuous variables. Variables that were shown to be significant in the initial analysis were further examined using multiple regression. Finally, Kaplan-Meier survival curves were analyzed for the selected variables, and the log-rank test was used to determine whether there were significant differences in the survival curves. For the analysis of DFS, the variables used in the OS analysis were applied, and a machine learning approach was employed using the Random Forest Classifier method. The Random Forest model was implemented by constructing multiple decision trees during the training phase, with the final prediction being the average output (regression) of the individual trees. The process involved generating several bootstrap samples from the original dataset, with each sample used to train a distinct decision tree. At each node, a random subset of features was chosen, and the optimal split was determined by minimizing prediction error, typically using mean squared error for regression tasks. This method allowed the model to capture different patterns in the data without over-relying on any single feature or subset. The model was trained using 80% of the data, while the remaining 20% was reserved for testing to evaluate its performance. Feature importance scores were subsequently calculated for all variables to determine their influence on the model’s predictions. A two-sided *P* value of < 0.05 was considered statistically significant. All statistical analyses were performed using the R software package, version 4.2.1. Machine learning analyses, including the implementation of the Random Forest algorithm, were conducted using the Python programming language with the scikit-learn library (version 0.24.2).

## Results

**Fig. 1:**
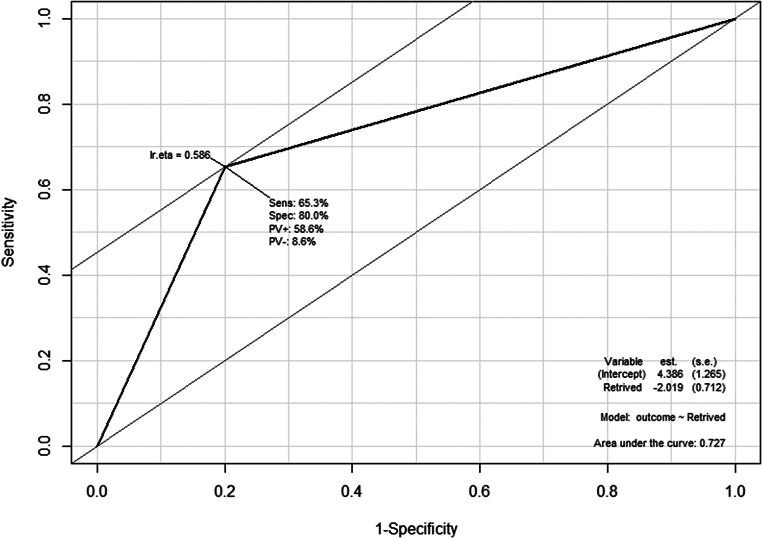
Receiver Operating characteristic curve for predicting outcome based on retrieved lymph nodes

**Fig. 2:**
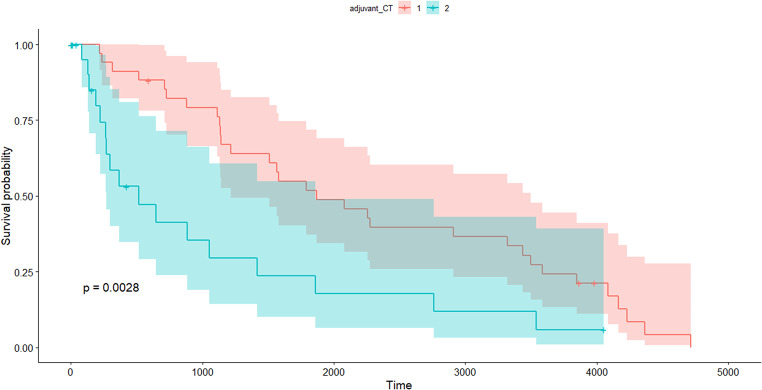
Kaplan-Meier survival curves comparing overall survival based on adjuvant chemotherapy administration

**Fig. 3:**
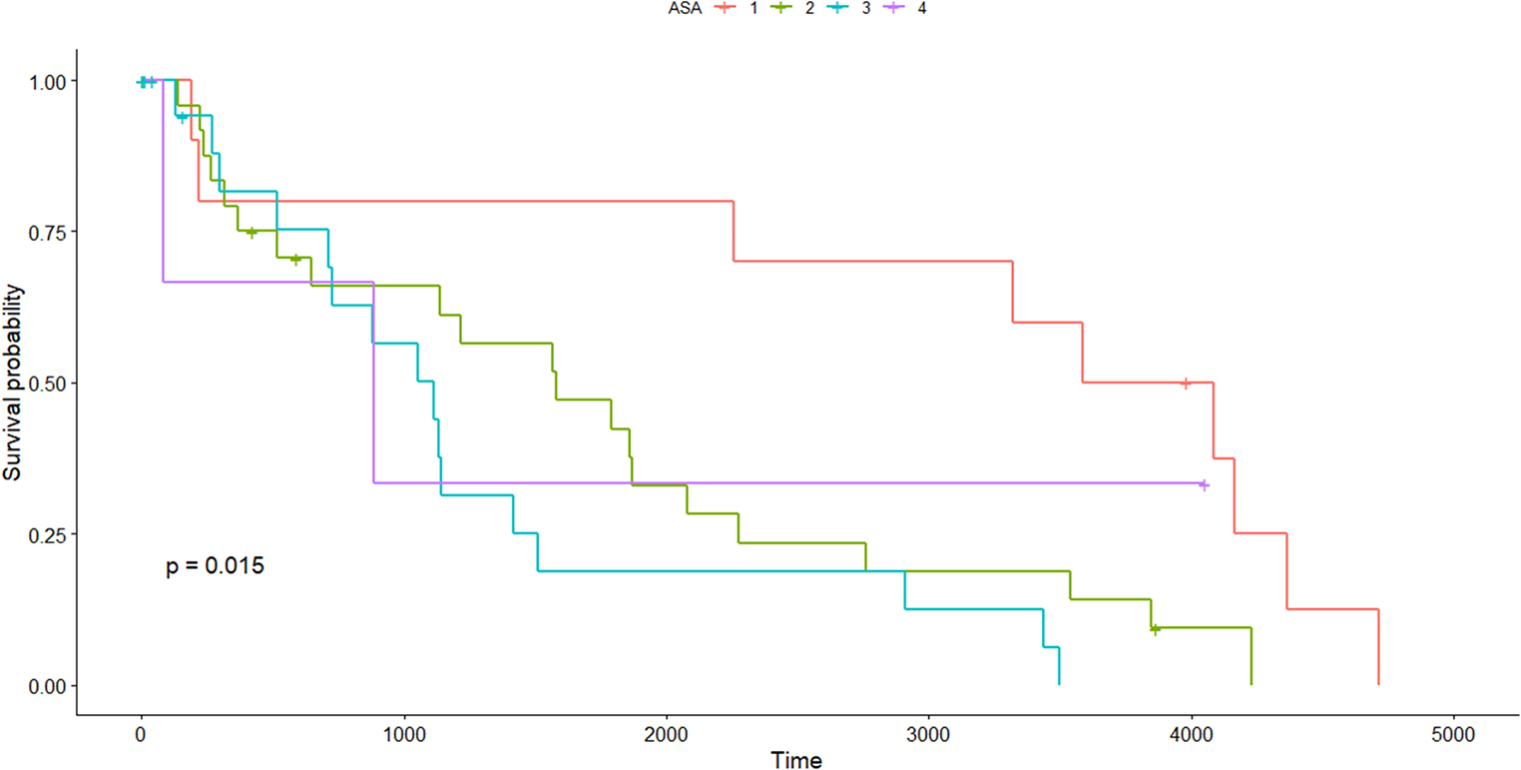
Kaplan-Meier survival curves comparing overall survival based on American Society of Anesthesiologists physical status

A total of 64 patients were included in the study. Among them, 46 patients presented with free colorectal perforation leading to generalized peritonitis. Of the remaining 16 patients, 7 presented with localized perforation at the tumor site, while the other 9 had complete obstruction without any perforation. (Table 1). All procedures were performed using an open surgical approach. Patients who died within 90 days had a higher incidence of preoperative circulatory shock (53.3% vs. 4.1%, *P =* 0.000), and both their i-SOFA (initial Sequential Organ Failure Assessment) and p-SOFA (postoperative Sequential Organ Failure Assessment) scores were significantly higher (2.80 vs. 0.53, *P =* 0.000; 5.67 vs. 1.76, *P =* 0.013, respectively). Survivors were more likely to have had 12 or more LNs retrieved during surgery (65.3% vs. 20%, *P =* 0.010), while complications were more frequently observed in non-survivors (66.7% vs. 32.7%, *P =* 0.040). There were no significant differences between the groups in terms of gender, age, presence of free perforation, lesion location, American Society of Anesthesiologists (ASA) physical status, or whether an anastomosis was performed. Multivariate analysis was conducted on the variables that showed differences between the groups, and only the number of retrieved LNs demonstrated a significant difference (*P =* 0.010). The ROC curve showed that the model performs reasonably well, with an AUC of 0.727, indicating a moderate ability to discriminate between outcomes based on the number of retrieved LNs (Fig. 1). Table 2 outlines aspects related to OS as part of the oncologic outcomes. Younger patients (*P =* 0.001), those with lower ASA scores (*P =* 0.000), absence of distant metastasis (*P =* 0.002), adjuvant CTx (*P =* 0.000), and lower LNR (*P =* 0.030) were associated with a longer survival. Multivariate analysis identified two factors that were significantly associated with OS: ASA physical status and adjuvant CTx. Specifically, differences were observed in patients with ASA II and III compared to ASA I, whereas no significant difference could be determined for patients with ASA IV. Figure 2 illustrates the OS of patients, stratified by whether they received adjuvant CTx. The red line represents patients who received adjuvant chemotherapy, while the blue line represents those who did not. The x-axis indicates time in days, and the y-axis represents survival probability. Patients who received adjuvant CTx demonstrated a higher survival probability over time compared to those who did not. The shaded areas around each curve depict the confidence intervals, with the red group consistently showing a higher survival rate. For the 5-year survival rate, it was calculated to be approximately 50% for patients who received adjuvant CTx, and around 25% for those who did not. Figure 3 depicts the OS of patients categorized by ASA physical status. Patients with ASA I had the highest survival, followed by ASA II and ASA III, with progressively lower survival probabilities. The curve for ASA IV shows an intermediate trend, but no statistically significant differences were observed for this group.

**Table 1:** Comparison of demographic, clinical, surgical, and pathological differences among patients based on 90-day survival

	Total (*N* = 64)	Non-survivor (*N* = 15)	Survivor (*N* = 49)	*P* ^a^	*P* ^b^	CI
Gender (N, %)				0.970		
Male	36 (56.3%)	9 (60%)	27 (55.1%)			
Female	28 (43.8%)	6 (40%)	22 (44.9%)			
Age (years)	67.33 ± 14.49	70.07 ± 12.99	66.49 ± 14.95	0.580		
Free colorectal perforation (N, %)	46 (71.9%)	11 (73.3%)	35 (71.4%)	1.000		
Location of the lesion (N, %)				0.080		
Right side colon cancer	22 (34.4%)	4 (26.7%)	18 (36.7%)			
Left side colon cancer	35 (54.7%)	7 (46.7%)	28 (57.1%)			
Rectal cancer	7 (10.9%)	4 (26.7%)	3 (6.1%)			
ASA (N, %)				0.300		
I	10 (15.6%)	1 (6.7%)	9 (18.4%)			
II	27 (42.2%)	5 (33.3%)	22 (44.9%)			
III	23 (35.9%)	7 (46.7%)	16 (32.7%)			
IV	4 (6.2%)	2 (13.3%)	2 (4.1%)			
Operation (N,%)				0.580		
Resection and anastomosis	36 (56.3%)	7 (46.7%)	29 (59.2%)			
Resection and diversion	28 (43.8%)	8 (53.3%)	20 (40.8%)			
Circulatory shock (N, %)	10 (15.6%)	8 (53.3%)	2 (4.1%)	0.000	0.200	(-1.40, 8.36)
i-SOFA (score)	1.06 ± 1.90	2.80 ± 3.05	0.53 ± 0.89	0.000	0.190	(-1.64, 0.21)
p-SOFA (score)	2.67 ± 3.20	5.67 ± 4.85	1.76 ± 1.70	0.013	0.880	(-0.60, 0.76)
Retrived lymph node (N,%)				0.010	0.010	(-5.97, -1.09)
>= 12	35 (54.7%)	3 (20%)	32 (65.3%)			
< 12	29 (45.3%)	12 (80%)	17 (34.7%)			
Length of ICU stay (days)	4.64 ± 4.76	5.53 ± 5.48	4.37 ± 4.55	0.380		
Length of hospital stay (days)	19.16 ± 9.94	15.93 ± 9.88	20.14 ± 9.84	0.170		
Post-opertive complications, classified as Clavien-Dindo class III or higher (N,%)	26 (40.6%)	10 (66.7%)	16 (32.7%)	0.040	0.520	(-1.22, 2.86)

Values are presented as number (%) for categorical variables, or mean ± standard deviation for continuous variables

^a^For categorical variables, the Fisher’s exact tests or chi-squres tests were employed and for continuous variables, the Wilcoxon tests were used

^b^Multivariate logistic regression analysis for the variables that showed significance on univariate analysis

CI, confidence interval; ASA, American society of anesthesiologists physical status classification; i-SOFA, initial sequential organ failure assessment; p-SOFA, postoperative sequential organ failure assessment; ICU, intensive care unit

**Table 2:** Comprehensive analysis of determinants influencing overall survival

	*P* ^a^	*P* ^b^
Gender	0.140	
Age	0.001	0.502
Free colorectal perforation	0.132	
Location of the lesion	0.116	
ASA^c^	0.000	
II		0.033
III		0.011
IV		0.751
Type of operation	0.444	
Retrived lymph node^4^	0.757	
Distant metastasis	0.002	0.219
Adjuvant chemotherapy	0.000	0.011
LNR	0.030	0.216

^a^ANOVA on categorical variables, and linear regression for continuous variables

^b^Multiple regression for statistically significant factors on univariate analysis

^c^For multivariate analysis, ASA II, III, and IV scores were each compared with ASA I score

^d^Divided into 12 or more and less than 12

ASA, American society of anesthesiologists physical status classification; LNR, lymph node ratio

**Fig. 4:**
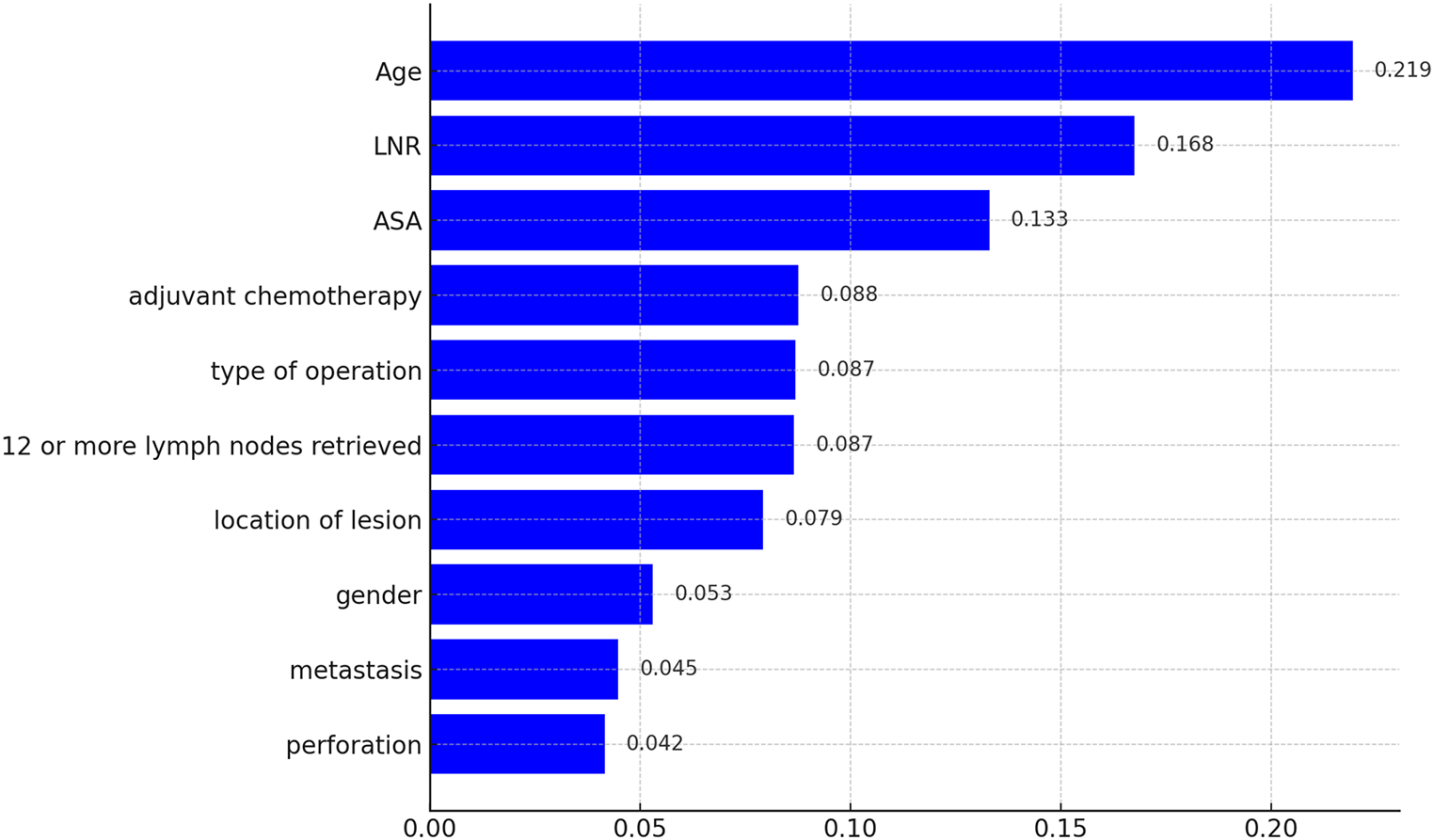
Feature importance in predicting cancer recurrence within 3 years using random forest model

Out of a total of 64 patients, 33 patients, corresponding to 51%, experienced recurrence within 3 years. The Random Forest model achieved an accuracy of 65% in predicting 3-year recurrence (Fig. 4). More specifically, in predicting the absence of recurrence, it demonstrated a precision of 0.69 and a recall of 0.75. The analysis of feature importance revealed that age was the most significant predictor, contributing 21.9% to the model’s decision-making process, followed by LNR with 16.8%. Based on these results, we applied a logistic regression model to calculate the quantitative impact of LNR and age on recurrence rates. The analysis revealed that for every 0.01-unit increase in LNR, the recurrence rate increased by approximately 1.025 times (2.5%), while for every 1-year increase in age, the recurrence rate increased by approximately 1.035 times (3.5%).

## Discussion

In the context of emergency surgery, the primary objective often centers on preserving life. However, in emergent CRC operation, surgeons must balance timely and rapid resuscitation, particularly for patients who are hemodynamically unstable, against the potential benefits of a more extensive oncologic resection. Generally, emergency surgery has been proven to entail higher mortality rates, complication rates, reoperation, and readmission compared to elective procedures [10], but the evidence regarding this relationship in emergent operations for CRC patients appears to be conflicting. Some researchers have observed that patients requiring emergent operations face higher rates of cancer-specific and overall mortality, even when curative resections are performed [11]. In contrast, other investigations indicate that, despite an increased risk of perioperative complications in the emergency setting, long-term outcomes can be comparable to those achieved with elective procedures [12, 13]. Notably, when emergency resections adhere to radical oncologic principles and aim for curative intent, survival rates may approximate those of elective surgery, particularly if tumor stages are matched [14]. However, not all emergent CRC operations can fully adhere to radical oncologic principles, and there is a paucity of research specifically examining prognostic determinants in patients who present with life threatening complications and require emergency surgery, where a curative resection may not always be feasible. In an effort to address this gap, our study targeted this particular cohort of emergent CRC patients, from which we identified the following key findings: (1) Patients who had 12 or more lymph nodes retrieved had a higher probability of 90-day survival; (2) Patients who received adjuvant CTx and those with lower ASA physical status had longer OS; and, (3) In predicting the likelihood of recurrence within 3 years, age (21.6%) and LNR (16.8%) were identified as the most important factors.

In clinical practice, surgeons frequently express excessive concern regarding factors traditionally perceived to predict poor clinical outcomes [15–17], including free colorectal perforation, advanced age, unfavorable physical status, preoperative shock, and elevated SOFA scores. We speculated that such tendencies might prompt surgeons, in complicated CRC emergencies, to abandon potentially feasible oncologic resections in favor of more minimal procedures, even when a more comprehensive surgical approach could have been viable. However, these variables were not significantly associated with 90 day survival in our stuy. In contrast to these traditional prognostic indicators, the only factor that significantly influenced 90-day survival was the number of retrieved LNs. Specifically, retrieving more than 12 LNs may indicate the patient’s hemodynamic stability, both preoperatively and intraoperatively, enabling meticulous dissection. The retrieval of more than 12 LNs is crucial for accurate oncologic staging [18]; however, it might be not the mere act of retrieving 12 or more LNs that directly enhances short-term prognosis. Rather, it can be the patient’s underlying condition, which permits the retrieval of this number of LNs, that is likely to have a more significant impact on short-term outcomes. Given that the number of retrieved lymph nodes is strongly influenced by the patient’s condition both before and during surgery, it is more prudent to evaluate each patient’s dynamic clinical status and intra-abdominal findings rather than predetermine the surgical plan, thereby guiding individualized decision-making. Furthermore, these findings indicate that it may not be necessary to preemptively restrict the surgical approach to a minimal procedure solely on the basis of each traditional prognostic factor.

Advanced cancer stage [19] and perforated CRC [20, 21] have been identified as poor prognostic factors for oncologic outcomes in emergent CRC operations. In a study involving patients without perforated CRC, adverse prognostic indicators included advanced age (over 75), right sided tumor location, an ASA score of 3 or higher, and LN metastasis [22]. Notably, studies specifically addressing the prognostic importance of retrived LN counts and LNR in emergency CRC surgeries are limited; instead, evidence from elective operations indicates that patients with fewer than 17 resected LNs [23] and those with an LNR of 18.2% or higher exhibit reduced OS and DFS [24]. Our study’s findings, while distinct from prior research, are based on a focused cohort of clinically unstable patients, necessitating a rapid response and emergent operation for CRC. Consequently, low ASA physical status and adjuvant CTx were confirmed to improve OS through multivariable logistic regression and Kaplan-Meier survival analysis. Furthermore, in predicting recurrence within three years, sequential statistical and machine learning analyses indicated that age and the LNR provided reliable predictive value. The analysis demonstrated that an increment of 0.01 unit in the LNR was associated with a roughly 1.025-fold increase in the recurrence rate. Additionally, each additional year in age corresponded to an approximate 1.035-fold increase in recurrence rate, reflecting a 3.5% rise. However, in contrast to previous studies, our study did not identify distant metastasis (indicating an advanced cancer stage) or free colorectal perforation as significant prognostic factors for OS or DFS. These results likely stem from incorporating the interrelationships among variables, particularly given that adjuvant CTx emerged as a predictor of OS. Consequently, the individual importance of free colorectal perforation and distant metastasis may have been diminished when analyzed in this multifactorial context. The adjuvant CTx has been known to improve long-term outcomes in cases of perforated CRC and is recommended for patients with stage III and high-risk stage II CRC, with perforation recognized as a significant high-risk factor in stage II cases [25, 26]. Nonetheless, not all patients undergoing emergent CRC operations appear to be suitable candidates for adjuvant CTx, particularly those with a low performance status. Even with optimal postoperative management, these patients may ultimately be unable to receive CTx, a point underscored by our observation that ASA physical status independently predicts OS. However, certain factors such as nutrition, infection control, or postoperative complications may still be modifiable. One study has indicated that minimizing postoperative complications can improve survival and reduce recurrence rates [27], underscoring the importance of precise surgical technique and diligent perioperative management. We propose that by optimizing postoperative management including nutritional support and fostering patient recovery in accordance with each individual’s capacity, a larger proportion of patients could become eligible for adjuvant chemotherapy.

During the study period, we endeavored to perform the most comprehensive oncologic surgery feasible for each patient, coupled with evidence-based perioperative management. However, the absence of a dedicated control group rendered direct comparisons of our outcomes impossible. Instead, we conducted an indirect comparison with national cancer registry data [28]. By the end of the study, 46 of the 64 patients included had died. Because the observation periods ranged from 3 to 15 years, a precise 5 year OS could not be calculated; however, we estimated an approximate OS of 28%. Although this figure is lower than the 74.3% 5 year survival reported by our national registry in 2021, it appears comparable to the 20.3% rate for patients with distant disease. These findings should be interpreted in light of several limitations, including the study’s retrospective design and relatively small sample size. We sought to mitigate these constraints through rigorous, stepwise statistical analyses and by incorporating machine learning methods to strengthen our results. Nonetheless, potential class imbalances (for example, fewer metastatic cases) and a limited range of collected variables may have curtailed the model’s capacity to generalize and detect recurrence patterns effectively.

In conclusion, of the factors identified as predictors of OS (ASA physical status and adjuvant CTx) and 3-year disease recurrence (age and LNR), ASA physical status and age lie largely beyond clinical control. However, our findings underscore the potential to improve outcomes through adjuvant CTx and by minimizing the LNR. While these metrics may partly reflect a patient’s underlying severity, we have observed that adjuvant CTx can often be administered successfully once meticulous postoperative management has stabilized the patient. Likewise, the LNR might be lowered by retrieving a greater number of LNs with a careful surgical technique. Collectively, these observations suggest that even within the emergent CRC setting, a deliberate focus on adjuvant CTx and comprehensive oncologic dissection can help optimize both short- and long-term outcomes.

## Data Availability

The data that support the findings of this study are not openly available due to reasons of sensitivity and are available from the corresponding author upon reasonable request. (jinbum21@catholic.ac.kr)

## References

[1] International Agency for Research on Cancer, World Health Organization (2020) Colon. Global Cnacer Observatory, Cancer Fact Sheet, Colon. https://gco.iarc.who.int/media/globocan/factsheets/cancers/8-colon-fact-sheet.pdf. Accessed 07 November 2024

[2] Kang DB, Shin CY, Lee JK, Park WC (2009) Multivariate analysis of the risk factors associated with complications and mortality after and emergency operation for obstructive, perforated colorectal Cancer. J Korean Soc Coloproctol 25:165–171. 10.3393/jksc.2009.25.3.165

[3] Kyllönen LE (1987) Obstruction and perforation complicating colorectal carcinoma. An epidemiologic and clinical study with special reference to incidence and survival. Acta Chir Scand 153:607–614 3434101

[4] Rabeneck L, Paszat LF, Li C (2006) Risk factors for obstruction, perforation, or emergency admission at presentation in patients with colorectal cancer: a population-based study. Am J Gastroenterol 101:1098–1103. 10.1111/j.1572-0241.2006.00488.x 16573783 10.1111/j.1572-0241.2006.00488.x

[5] Pisano M, Zorcolo L, Merli C et al (2018) 2017 WSES guidelines on colon and rectal cancer emergencies: obstruction and perforation. World J Emerg Surg 13:36. 10.1186/s13017-018-0192-3 30123315 10.1186/s13017-018-0192-3 PMC6090779

[6] van Hooft JE, Veld JV, Arnold D et al (2020) Self-expandable metal stents for obstructing colonic and extracolonic cancer: European society of Gastrointestinal endoscopy (ESGE) Guideline - Update 2020. Endoscopy 52:389–407. 10.1055/a-1140-3017 32259849 10.1055/a-1140-3017

[7] Cianchi F, Palomba A, Boddi V et al (2002) Lymph node recovery from colorectal tumor specimens: recommendation for a minimum number of lymph nodes to be examined. World J Surg 26:384–389. 10.1007/s00268-001-0236-8 11865379 10.1007/s00268-001-0236-8

[8] Swanson RS, Compton CC, Stewart AK, Bland KI (2003) The prognosis of T3N0 colon cancer is dependent on the number of lymph nodes examined. Ann Surg Oncol 10:65–71. 10.1245/aso.2003.03.058 12513963 10.1245/aso.2003.03.058

[9] Clavien PA, Barkun J, de Oliveira ML et al (2009) The Clavien-Dindo classification of surgical complications: five-year experience. Ann Surg 250:187–196. 10.1097/SLA.0b013e3181b13ca2 19638912 10.1097/SLA.0b013e3181b13ca2

[10] Mullen MG, Michaels AD, Mehaffey JH et al (2017) Risk associated with complications and mortality after urgent surgery vs elective and emergency surgery: implications for defining quality and reporting outcomes for urgent surgery. JAMA Surg 152:768–774. 10.1001/jamasurg.2017.0918 28492821 10.1001/jamasurg.2017.0918 PMC5710495

[11] McArdle CS, Hole DJ (2004) Emergency presentation of colorectal cancer is associated with poor 5-year survival. Br J Surg 91:605–609. 10.1002/bjs.4456 15122613 10.1002/bjs.4456

[12] Boeding JRE, Ramphal W, Rijken AM et al (2021) A systematic review comparing emergency resection and staged treatment for curable obstructing Right-Sided Colon cancer. Ann Surg Oncol 28:3545–3555. 10.1245/s10434-020-09124-y 33067743 10.1245/s10434-020-09124-y

[13] Mohan HM, Evans MD, Larkin JO, Beynon J, Winter DC (2013) Multivisceral resection in colorectal cancer: a systematic review. Ann Surg Oncol 20:2929–2936. 10.1245/s10434-013-2967-9 23666095 10.1245/s10434-013-2967-9

[14] Biondo S, Martí-Ragué J, Kreisler E et al (2005) A prospective study of outcomes of emergency and elective surgeries for complicated colonic cancer. Am J Surg 189:377–383. 10.1016/j.amjsurg.2005.01.009 15820446 10.1016/j.amjsurg.2005.01.009

[15] Xu X, Dong HC, Yao Z, Zhao YZ (2020) Risk factors for postoperative sepsis in patients with Gastrointestinal perforation. World J Clin Cases 8:670–678. 10.12998/wjcc.v8.i4.670 32149051 10.12998/wjcc.v8.i4.670 PMC7052561

[16] Shin R, Lee SM, Sohn B et al (2016) Predictors of morbidity and mortality after surgery for intestinal perforation. Ann Coloproctol 32:221–227. 10.3393/ac.2016.32.6.221 28119865 10.3393/ac.2016.32.6.221 PMC5256250

[17] Sumi T, Katsumata K, Katayanagi S et al (2014) Examination of prognostic factors in patients undergoing surgery for colorectal perforation: a case controlled study. Int J Surg 12:566–571. 10.1016/j.ijsu.2014.03.021 24709571 10.1016/j.ijsu.2014.03.021

[18] Baxter NN, Virnig DJ, Rothenberger DA, Morris AM, Jessurun J, Virnig BA (2005) Lymph node evaluation in colorectal cancer patients: a population-based study. J Natl Cancer Inst 97:219–225. 10.1093/jnci/dji020 15687365 10.1093/jnci/dji020

[19] Lee HJ, Oh JH, Lee JN et al (2006) Prognostic factors associated with surgical mortality conferred by emergency operation in colorectal Cancer. J Korean Soc Coloproctol 22:301–307

[20] Zamaray B, van Velzen RA, Snaebjornsson P, Consten ECJ, Tanis PJ, van Westreenen HL (2023) Outcomes of patients with perforated colon cancer: A systematic review. Eur J Surg Oncol 49:1–8. 10.1016/j.ejso.2022.08.008 35995649 10.1016/j.ejso.2022.08.008

[21] Yang KM, Jeong MJ, Yoon KH, Jung YT, Kwak JY (2022) Oncologic outcome of colon cancer with perforation and obstruction. BMC Gastroenterol 22:247. 10.1186/s12876-022-02319-5 35570293 10.1186/s12876-022-02319-5 PMC9107675

[22] Manceau G, Voron T, Mege D et al (2019) Prognostic factors and patterns of recurrence after emergency management for obstructing colon cancer: multivariate analysis from a series of 2120 patients. Langenbecks Arch Surg 404:717–729. 10.1007/s00423-019-01819-5 31602503 10.1007/s00423-019-01819-5

[23] Wei R, Zheng Z, Li Q et al (2024) Prognostic and predictive value of examined lymph node count in stage III colorectal cancer: a population based study. World J Surg Oncol 22:155. 10.1186/s12957-024-03404-7 38872183 10.1186/s12957-024-03404-7 PMC11170906

[24] Hai ZX, Peng D, Li ZW, Liu F, Liu XR, Wang CY (2024) The effect of lymph node ratio on the surgical outcomes in patients with colorectal cancer. Sci Rep 14:17689. 10.1038/s41598-024-68576-4 39085386 10.1038/s41598-024-68576-4 PMC11291744

[25] Asano H, Kojima K, Ogino N, Fukano H, Ohara Y, Shinozuka N (2017) Postoperative recurrence and risk factors of colorectal cancer perforation. Int J Colorectal Dis 32:419–424. 10.1007/s00384-016-2694-3 27796497 10.1007/s00384-016-2694-3

[26] Benson AB 3rd, Schrag D, Somerfield MR et al (2004) American society of clinical oncology recommendations on adjuvant chemotherapy for stage II colon cancer. J Clin Oncol 22:3408–3419. 10.1200/jco.2004.05.063 15199089 10.1200/JCO.2004.05.063

[27] Law WL, Choi HK, Lee YM, Ho JW (2007) The impact of postoperative complications on long-term outcomes following curative resection for colorectal cancer. Ann Surg Oncol 14:2559–2566. 10.1245/s10434-007-9434-4 17522945 10.1245/s10434-007-9434-4

[28] Korea Central Cancer Registry, National Cancer Center (2023) Annual report of cancer statistics in Korea in 2021. Ministry of Health and Welfare. https://ncc.re.kr/cancerStatsList.ncc?searchKey=total%26searchValue=%26pageNum=1

